# Primary multifocal osseous Hodgkin’s lymphoma with spinal involvement

**DOI:** 10.1093/omcr/omaf223

**Published:** 2026-01-25

**Authors:** Nouman Nawaz Ali Nathani, Lavita Kumari, Natasha Ali

**Affiliations:** Department of Pathology and Laboratory Medicine, The Aga Khan University Hospital, Stadium Road, P.O. Box 3500, Karachi 74800, Pakistan; Department of Pathology and Laboratory Medicine, The Aga Khan University Hospital, Stadium Road, P.O. Box 3500, Karachi 74800, Pakistan; Professor Haematology, Department of Pathology and Laboratory Medicine, The Aga Khan University Hospital, Stadium Road, P.O. Box 3500, Karachi 74800, Pakistan

**Keywords:** primary osseous Hodgkin lymphoma, ABVD, spinal involvement

## Abstract

A 40-year-old male presented with progressive backache; his workup revealed primary multifocal osseous Hodgkin’s lymphoma (PMOHL), which is a very rare primary presentation of Hodgkin’s lymphoma (HL). The common presenting symptoms include localized bone pain and tenderness with or without fever, weight loss or drenching night sweats. Given the rarity of the spine being affected in PMOHL and its symptoms overlapping with conditions like infectious or malignant causes, this case adds meaningful insight to existing literature so that delays in the diagnosis can be avoided. This case also signifies the need of repeat biopsy to prevent unnecessary delays and polypharmacy. Once diagnosis is made, adriamycin, bleomycin, vinblastine, and dacarbazine (ABVD) is still curative and cost-effective regimen in the cost constraint part of the world.

## Introduction

Hodgkin’s lymphoma (HL) originates from B lymphocytes with the hallmark feature of the presence of Reed-Sternberg cells [1]. Bone involvement may occur in around 10% to 20% of cases. [2]. In contrast, primary multifocal osseous Hodgkin’s lymphoma (PMOHL) arises directly in bone tissue, without lymph node or visceral involvement, and the frequent sites include pelvic bones, humerus, vertebra, and femur [2, 3]. The common presentation is persistent, localized bone pain, often accompanied by swelling and tenderness in the affected areas [1, 3]. Radiological and histopathological features of PMOHL can show mixed osteosclerosis/osteolysis, which resemble conditions like eosinophilic granuloma, tuberculosis, multiple myeloma, or osteomyelitis, making misdiagnosis a common pitfall [1, 2]. Positron Emission Tomography/Computed Tomography (PET/CT) is the modality of choice due to response-adapted treatment of HL, which avoids both under- and over-treatment [4, 5]. We are highlighting the diagnostic challenges and clinical management of this rare entity.

**Figure 1:**
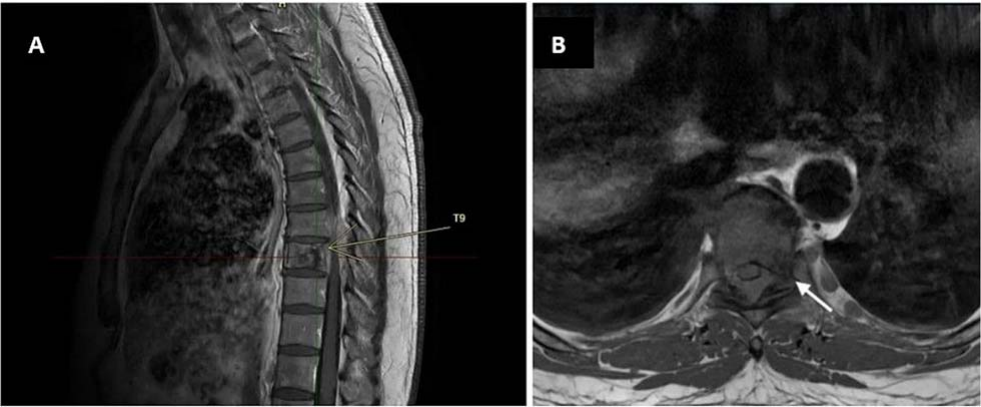
Figure 1 Contrast-enhanced MRI of the thoracic spine. Sagittal view (A, yellow arrow) and axial view (B, white arrow) show an abnormal signal in the T9 vertebra.

## Case presentation

A 40-year-old male with a history of hypertension, controlled with amlodipine 5 mg daily, experienced increasing back pain in his mid-thoracic region starting in September 2023. Initially intermittent, the pain worsened with specific postures, like riding a bike or lying flat, and eventually became constant and more severe. He also reported low-grade fevers, without chills or rigors, that subsided with over-the-counter antipyretics. Family history was negative for cancer, no history of recent travel or substance use, or known infections. On clinical examination, localized tenderness and mild restriction in spinal movement were observed, without lymphadenopathy or visceromegaly.

He was referred to a neurosurgeon; the MRI showed diffuse abnormal signals in the T9 vertebral body and posterior elements (Fig. 1), along with an enhancing soft tissue mass extending into the epidural space and neural foramina. This resulted in spinal cord and radicular compression at the T8-T9 and T9-T10 levels, suggestive of a metastatic process. A CT-guided biopsy of the T9 lesion revealed only nonspecific inflammation. Therefore, T8–T9 laminectomy was performed in October 2023.

**Figure 2:**
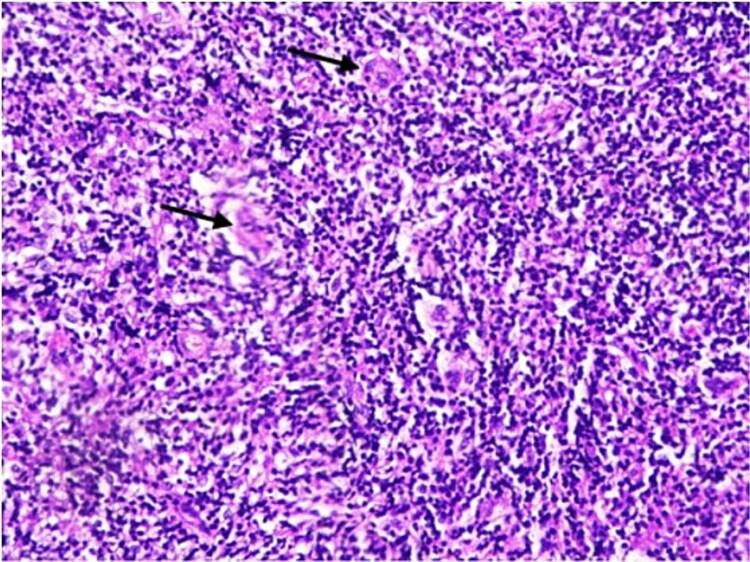
Scattered large Reed-Sternberg cells (arrows) with prominent nucleoli in a mixed inflammatory background (H&E, 200x).

**Figure 3:**
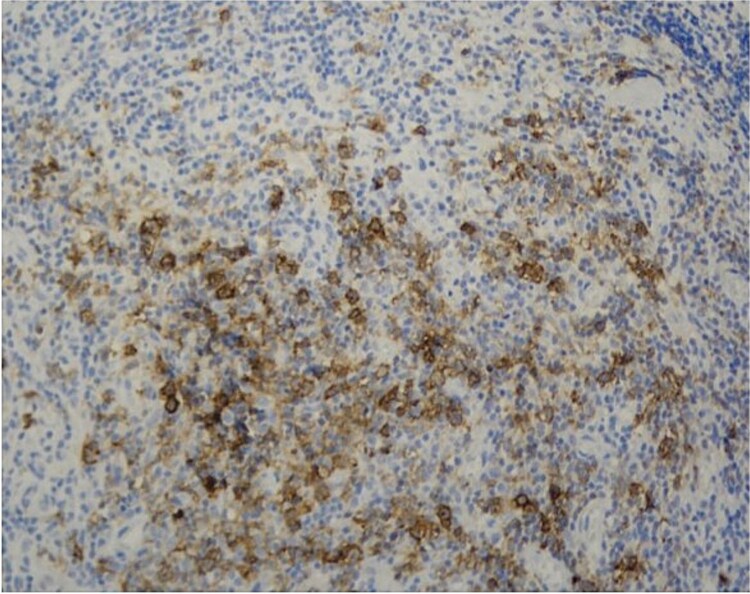
CD30 Positivity in Reed-Sternberg cells (CD30, 200x).

Histopathology showed fibro-collagenous tissue infiltrated by mature lymphocytes, histiocytes, eosinophils, and neutrophils. Importantly, occasional large atypical mononuclear cells were noted (Fig. 2). Immunohistochemical staining indicated that these atypical cells were negative for LCA, CD20, and EBV; weakly positive for PAX5; and positive for CD30 (Fig. 3), with occasional cells staining positive for CD15. These morphological and immunohistochemical findings were consistent with a diagnosis of classical Hodgkin lymphoma. The patient was referred to the hematology clinic for further management.

A PET-CT revealed surgical changes in the paraspinal region and hypermetabolic sclerotic lesions at T9 (SUV max 12.9), the T8 left pedicle (SUV max 5.1), and the glenoid of the left scapula (SUV max 5.9). No nodal or visceral disease was identified; hence, stage IV disease with the Hasenclever.

**Figure 4:**
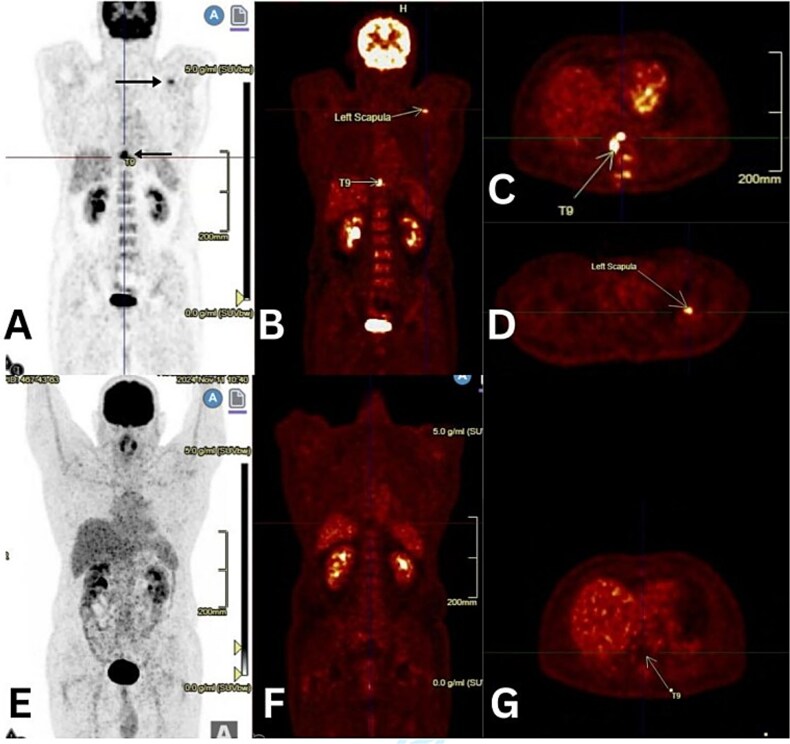
Figure 4 (A-D) initial PET/CT images showing abnormal uptake at the T9 vertebra and left scapula (black and yellow arrows). (E-G) end-of-treatment PET/CT images showing resolution of abnormal uptake.

International Prognostic Score (IPS) of 3. Bone trephine was suboptimal. After hepatitis B, C, human immunodeficiency virus status, renal and hepatic function tests, echocardiography, and pulmonary function tests, he received six cycles of ABVD chemotherapy, to which the end-of-treatment PET/CT showed a complete metabolic response (Deauville 5PS: 01) (Fig. 4).

## Discussion

Primary multifocal osseous Hodgkin’s lymphoma (PMOHL) is an uncommon manifestation of HL, involving multiple bones without lymph node or organ involvement for a period of at least six months, and there is always a possibility that clinicians may not reach the correct diagnosis [3, 6]. PMOHL arises directly in bone tissue, without lymph node or visceral involvement for at least six months from the onset, and the frequent sites include pelvic bones, humerus, vertebra, and femur [2, 3]. When it involves the vertebral column, the disease can be mistaken for osteomyelitis or Pott's disease [3], which emphasizes the need for early tissue diagnosis and a multidisciplinary approach as depicted in our case.

MRI showed a lytic lesion in T9 with epidural extension, producing spinal cord compression. Such findings more commonly point toward metastasis or spinal tuberculosis rather than HL. Further PET-CT confirmed metabolically active lesions in the T8 vertebra and scapula, with no nodal involvement. Of note, our patient had spinal cord compression, a rare presenting feature of HL and seen in only ~ 0.2% of cases, without lymphadenopathy, adding to the diagnostic challenge [7].

The initial CT-guided biopsy failed to detect neoplastic cells, revealing only nonspecific inflammation, which is a frequent issue in PMOHL [8], likely due to sparse Reed-Sternberg cells embedded in an inflammatory background [1, 2]. The diagnosis became clearer only after a second biopsy obtained via surgical laminectomy, as suggested by Luo W et al [6]. Immunohistochemical markers (CD30+, weak PAX5+, CD15 occasional+, CD20-, LCA-) confirmed PMOHL [7, 9].

The patient’s treatment with ABVD chemotherapy yielded excellent results. Up to now, at the 1-year follow-up, there is no sign of disease relapse. This regimen remains a standard for HL and has shown promising outcomes in rare presentations like PMOHL [6, 10].

This case points to the fact that the diagnosis of PMOHL is difficult and easily gets confused with common conditions like Pott’s disease or metastases. In this patient’s case, the unusual presentation and inconclusive first biopsy led to an initial delay. The repeat biopsy was performed and the patient responded to ABVD chemotherapy, which remains an affordable and effective treatment option to date.

## Patient’s perspective

I had never experienced such a type of pain and misery. This illness started as a simple backache, which I ignored initially but made me worried once it started affecting my daily routine. I would suggest others not to take any non-specific symptoms for granted. I am now back to my routine, carrying out daily activities like I used to in the past.

## Data Availability

Not applicable.
